# Differential impact of behavioral, social, and emotional apathy on Parkinson's disease

**DOI:** 10.1002/acn3.626

**Published:** 2018-08-14

**Authors:** Yuen‐Siang Ang, Patricia L. Lockwood, Annika Kienast, Olivia Plant, Daniel Drew, Elitsa Slavkova, Marin Tamm, Masud Husain

**Affiliations:** ^1^ Nuffield Department of Clinical Neurosciences University of Oxford Oxford United Kingdom; ^2^ Department of Experimental Psychology University of Oxford Oxford United Kingdom; ^3^ Department of Experimental Psychology Wellcome Trust Centre for Integrative Neuroimaging University of Oxford Oxford United Kingdom

## Abstract

Apathy is highly prevalent in Parkinson's disease. New findings suggest the syndrome is multifaceted. Here, we investigate whether all aspects of apathy are equally affected in Parkinson's disease and whether different dimensions of apathy were associated with depression and anhedonia. On the Apathy Motivation Index, while behavioral apathy and social apathy were elevated, emotional motivation was relatively preserved in Parkinson's disease, although a few patients did show impaired emotional sensitivity. Behavioral and social, but *not* emotional, apathy was associated with depression and anhedonia. These findings suggest aspects of motivation can be selectively impaired in Parkinson's disease and may have implications for guiding treatment.

## Introduction

Apathy, a disorder of motivation characterized by reduced self‐initiated goal‐directed behavior,^1^ is very common in Parkinson's disease (PD) and associated with diminished quality of life.^2^, ^3^, ^4^ Theoretical accounts^5^ and new evidence^6^ suggest that rather than being a unitary construct, apathy is multidimensional, involving behavioral, cognitive, executive, social, and emotional domains. Despite the high prevalence of apathy in PD, it is unclear whether all domains are affected.^7^ Previous studies have yielded mixed results; both greater emotional apathy but preserved executive motivation^8^ and preserved emotional motivation but executive and initiation deficits^9^ have been reported. Moreover, apathy appears to have overlapping symptoms with other comorbid states associated with altered motivation, particularly depression^10^ and anhedonia.^11^ It remains to be established whether different apathy domains in PD relate specifically to these syndromes.

Here, we used the Apathy Motivation Index (AMI), a new self‐report measure of apathy, rigorously validated in a large sample of healthy people,^6^ to examine the multidimensional profile of apathy in PD. We also assessed how different apathy domains were related to depression and anhedonia. The AMI identifies apathy in *behavioral*,* social,* and *emotional* domains and was developed from work based on the Lille Apathy Rating Scale (LARS), a well‐validated structured interview of apathy in PD.^12^ The willingness to exert effort for reward correlates with the behavioral dimension of the AMI, whereas the willingness to put in effort for other people correlates with the social apathy dimension of the AMI,^13^ suggesting meaningfully behavioral correlates. Moreover, the AMI is quick and easy to administer.

We first established the reliability and validity of the AMI in PD by comparing measurement of apathy using the AMI to the LARS. Next, we assessed whether there are differences in apathy dimensions in patients with PD compared with healthy controls, and whether the different domains of apathy exhibit differential associations with depression and anhedonia.

## Methods

### Participants

A total of 102 patients with PD were recruited from Neurology clinics and 147 healthy age‐ and gender‐matched controls from a volunteer database (demographics in Table 1; inclusion and exclusion criteria in Data S1). All patients and controls had no history of other neurological or psychiatric conditions, gave written informed consent, and the study was approved by the local ethics committee.

**Table 1:** Participant demographics

	PD (*n* = 102)	HC (*n* = 147)	PD vs. HC
Age (years)	67.7 ± 8.1	66.1 ± 8.5	*P *>* *0.05
Gender (M:F)	79:23	104:43	*P *>* *0.05
UPDRS‐III^a^	27.0 ± 13.2	N/A	N/A
ACE^b^	89.4 ± 9.0	N/A	N/A
Disease duration (years)^c^	6.6 ± 3.9	N/A	N/A
LED (mg/24 h)^d^	540.8 ± 333.1	N/A	N/A
Education (years)^e^	14.6 ± 3.8	N/A	N/A

a Unified Parkinson's Disease rating scale (*N *=* *13 missing)

b Addenbrooke's cognitive examination (*N *=* *13 missing)

c Disease duration (*N *=* *2 missing)

d Levodopa equivalent dose (*N *=* *7 missing)

e Education (*N *=* *16 missing)

### Measures

Participants completed the Apathy Motivation Index (AMI),^6^ which assesses apathy in terms of behavioral activation (BA: tendency to self‐initiate goal‐directed behavior), social motivation (SM: level of engagement in social interactions), and emotional sensitivity (ES: feelings or affective responses), the Lille Apathy Rating Scale (LARS)^12^ (patients *N *=* *87), the Snaith–Hamilton Pleasure Scale (SHAPS)^14^ (patients *N *=* *84, controls *N *=* *67), and the Geriatric Depression Scale–Short Form (GDS‐15)^15^ (patients *n* = 80, controls *N *=* *87).

### Statistical analysis

Data were analyzed using SPSS v22.0, with *P *<* *0.05 two‐tailed. Dependent variables were compared using independent‐samples *t*‐test and mixed ANOVAs. Levene's test, Mauchly's test and Greenhouse–Geisser corrections were applied when necessary. Correlational comparisons were corrected for false discovery rate using the Benjamini–Hochberg procedure.

## Results

### AMI has good internal reliability, validity, and diagnostic accuracy in PD

**Figure 1:**
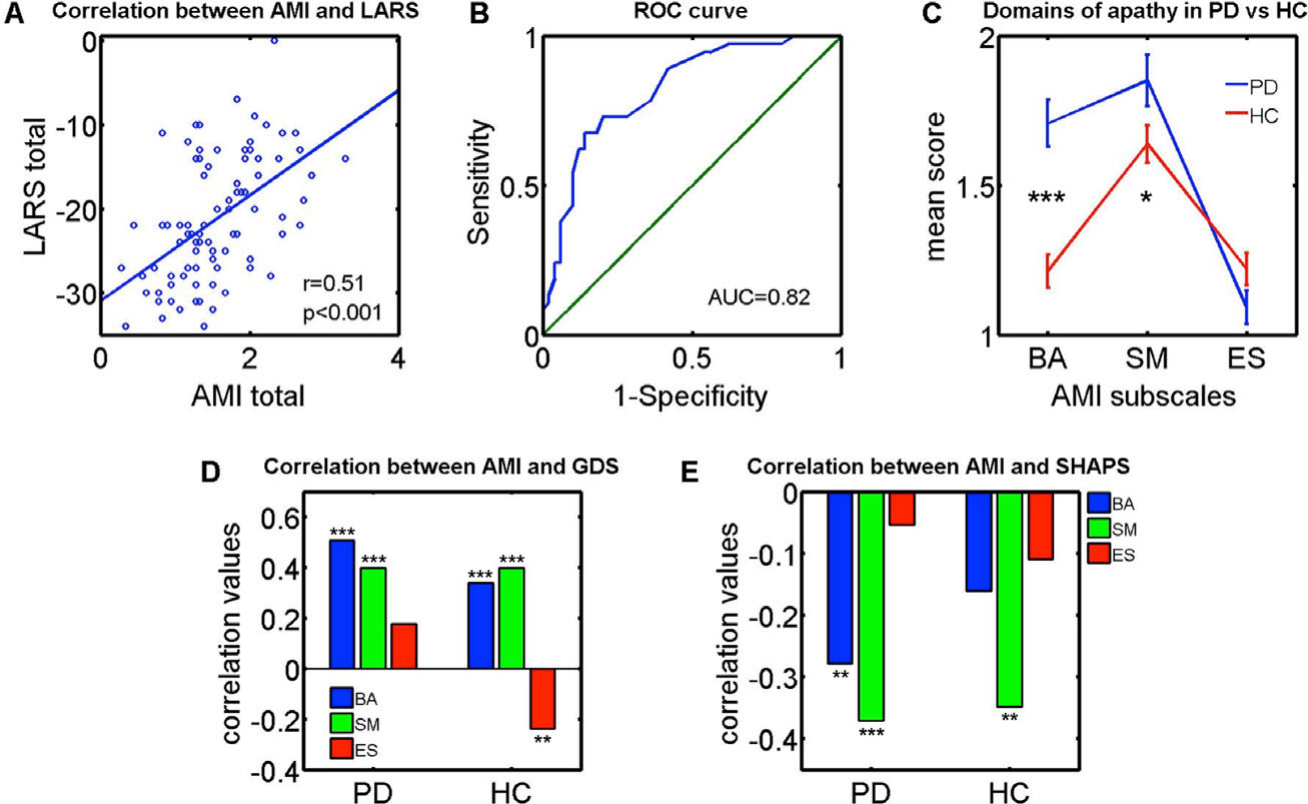
Apathy Motivation Index findings in Parkinson's disease. (A) There was a significant positive correlation between the AMI and LARS, an established clinical interview measure of apathy. (B) An ROC curve analysis performed by adopting the LARS as the gold standard found that the AMI has good diagnostic accuracy for clinical apathy. (C) PD patients exhibited significantly greater levels of behavioral and social apathy compared to age‐matched healthy controls, but there was no significant difference in emotional apathy between the two groups. (D) Behavioral activation (BA) and social motivation (SM) correlated positively with Geriatric Depression Scale‐15 (GDS) score in both PD and controls, indicating that individuals who were more behaviorally and socially apathetic were more likely to be depressed. Emotional sensitivity (ES), however, did *not* correlate with depression in PD but correlated negatively in controls. Comparison of correlation coefficients between patients and controls via Fisher's r‐to‐z transformation confirmed there was a significant difference between ES and depression, but not BA and SM. (E) BA and SM correlated negatively with Snaith–Hamilton Pleasure Scale (SHAPS) in PD: Patients who were behaviorally and socially more apathetic tended to be more anhedonic. However, there was no relationship with ES. In controls, only social apathy exhibited a significant correlation with anhedonia. Fisher's transformation showed no significant differences between patients and controls in correlation between anhedonia and the three apathy subscales. AMI, Apathy Motivation Index; LARS, Lille Apathy Rating Scale. *: p<0.05; **: p<0.01; ***: p<0.001

Cronbach's alpha values for AMI total and subscales were good (*α*_overall _= 0.86, *α*_BA _= 0.79, *α*_SM _= 0.80, *α*_ES _= 0.66). Moreover, AMI total score correlated positively with LARS overall score (*r *=* *0.51, *P *<* *0.001, Fig. 1A), demonstrating good construct validity. An ROC analysis was also conducted to evaluate the diagnostic accuracy of the AMI against the LARS. Thirty‐seven apathetic and 50 nonapathetic patients were identified based on the LARS cutoff of >−22, and the area under curve was 0.82, indicating good diagnostic accuracy (Fig. 1B). Furthermore, according to the AMI cutoff scores derived from Ang et al. (2017),^6^ 36 of all 102 patients with PD were apathetic on at least one subscale (Fig. S1). This not only provides a finer‐grained classification of apathy in terms of its multiple dimensions, but also reflects an overall prevalence rate of apathy that is consistent with the existing literature.^2^

### More behavioral and social apathy in PD

Patients had a higher total AMI score than controls (*t*(163) = 2.9, *P *=* *0.005), indicative of greater apathy overall. There was no effect of age, sex, cognition, years of education, disease duration, and levodopa equivalent doses on AMI total or individual subscale scores in PD (Data S2). Next, we examined whether patients have greater apathy in particular domains or were simply more apathetic in general.

A mixed ANOVA with group as the between‐subjects factor (patients vs. controls) and AMI subscales as within‐subjects factor (BA vs. SM vs. ES) revealed a significant interaction effect (*F*(1.8,448) = 14.1, *P *<* *0.001). Post hoc comparisons showed that overall PD patients exhibited higher levels of behavioral (*P *<* *0.001) and social (*P *<* *0.05) apathy relative to controls (Fig. 1C). Emotional apathy, however, did not differ between the two groups (*P *=* *0.11). However, there were six cases who showed pure deficits in emotional sensitivity but not the other domains (Fig. S1).

### Apathy domains are differentially associated with depression and anhedonia

Individuals who were more behaviorally and socially apathetic were more likely to be depressed. However, the emotional apathy subscale did *not* associate with depression in patients, although it was negatively correlated in controls (Fig. 1D; **PD:** *r*_BA _= 0.51, *P *<* *0.001, *r*_SM _= 0.40, *P *<* *0.001, *r*_ES_=0.18, *P *>* *0.05; **Controls:** *r*_BA _= 0.34, *P *=* *0.001, *r*_SM _= 0.40, *P *<* *0.001, *r*_ES _= −0.24, *P *<* *0.05). Comparison of correlation coefficients between patients and controls via Fisher's *r*‐to‐*z* transformation confirmed there was a significant difference in emotional apathy (*z*_BA _= 1.3, *P *>* *0.05; *z*_SM _= 0.03, *P *>* *0.05; *z* = 2.7, *P *= 0.007). This suggests that emotional apathy in PD was not related to depression, but healthy people who were more emotionally apathetic tended to be less depressed.

Patients who had higher levels of behavioral and social apathy were more anhedonic, but there was no association with emotional apathy. For controls, only the social apathy subscale had a significant negative correlation with SHAPS, indicating that healthy people who were more socially apathetic exhibited greater anhedonia (Fig. 1D; **PE:** *r*_BA _= −0.28, *P *=* *0.01, *r*_SM _= −0.37, *P *<* *0.001, *r*_ES _= −0.05, *P *>* *0.05; **Controls:** *r*_BA _= −0.16, *P *>* *0.05, *r*_SM _= −0.35, *P *=* *0.004, *r*_ES _= −0.11, *P *>* *0.05). Fisher's transformation showed no significant differences in anhedonia between groups (z_BA _= −0.8, *P *>* *0.05; z_SM _= −0.1, *P *>* *0.05; z = 0.4, *P *>* *0.05).

## Discussion

Here, we used the Apathy Motivation Index to investigate two critical issues in the assessment of PD. First, are all domains of apathy are affected in PD? and second, how are different domains of apathy associated with depression and anhedonia? PD patients exhibited greater behavioral and social apathy compared with healthy controls. However, overall, there was no difference in levels of emotional apathy, suggesting relatively preserved emotional motivation in PD, although a few patients did show pure emotional apathy. Moreover, the different domains of apathy exhibited differential associations with depression and anhedonia. In particular, while behavioral and social apathy were related to both depression and anhedonia, emotional apathy was not.

The current literature on emotional apathy in PD is mixed.^7^ Some researchers have reported that emotional facial recognition, which correlates with emotional apathy,^16^ is impaired in PD^17^, whereas others have found no impairments.^18^ Moreover, both intact emotional motivation^8^ and impaired emotional motivation have been reported albeit in a smaller sample.^9^ In our study, patients were significantly more apathetic behaviorally and socially but did not differ overall in emotional apathy compared with healthy controls. This finding is also consistent with clinical observations of reduced goal‐directed behavior being most frequently reported in apathetic PD patients and reduced emotion being least commonly found.^19^ Thus, it may be important to focus on which aspects of apathy—behavioral, social, or emotional—are affected in an individual when developing treatments, including psychological ones.

This pattern of multidimensional apathy may not be specific to PD, and future investigations could focus on other conditions in which apathy is prevalent such as Alzheimer's disease^20^ and schizophrenia.^21^ Theoretically, other psychiatric and neurological disorders might show different patterns of associations within the profile of apathy. For example, individuals with autism are predicted to have lower levels of social motivation^22^ but higher levels of emotional motivation.^23^ It might be important for future studies to document the profile of apathy in different disorders, which will be informative for treatment strategies.

In our study, patients with PD who were behaviorally and socially apathetic were also more depressed, but there was no association between emotional apathy and depression. However, in healthy controls, those who were more emotionally apathetic actually tended to be less depressed. Our finding that depression is negatively associated with ES in healthy controls but not in PD adds to existing studies regarding complex associations between apathy and depression.^10^ Importantly, this result suggests that different clinical approaches toward apathy in patients with PD compared to the general population might be needed.^24^ Our finding dovetails with other reports which show that apathy can be dissociated from depression in PD. Apathy frequently occurs in the absence of depression^10^, and factor analyses have revealed that the two represent discrete constructs.^10^ The findings presented here go further by highlighting the importance of considering the multidimensional nature of apathy when assessing relationships with depression.

Studies of anhedonia and apathy in PD are scarce and have also produced mixed results of both greater anhedonia in patients with apathy,^25^ and no association.^26^ We examined this relationship in a large sample and found that behavioral and social, but crucially not emotional, apathy correlated with anhedonia in PD. For controls, only social apathy exhibited a significant correlation with anhedonia even though a comparison of the correlations between groups showed no difference. This suggests that apathy and anhedonia are separable in the emotional domain, but have a close relationship in behavioral and social aspects.

Finally, our study revealed that the AMI is significantly correlated with the LARS and has good diagnostic accuracy for clinical apathy. This suggests that it may be a suitable alternative assessment of impaired motivation. Furthermore, the AMI is quicker and easier to administer as it does not require a clinician to be present.

## Conclusion

Here, we show that not all aspects of motivation are impaired in PD. Specifically, even though behavioral apathy and social apathy are elevated, emotional motivation appears to be preserved. Furthermore, in patients with PD behavioral and social apathy, symptoms are related to depression and anhedonia—whereas emotional apathy is not. Overall, similar relationships were found in controls, but in this group, there was a negative association between emotional apathy and depression. Together our findings may help in guiding the development of more effective, selective treatments for apathy in PD—including nonpharmacological ones aimed at different aspects of motivation—as well as assisting in our understanding of how apathy, anhedonia, and depression are related.

## Author Contributions

Y.A., P.L., and M.H. performed conceptualization. Y.A., P.L., and M.H. involved in methodology. Y.A. and P.L. carried out formal analysis. Y.A., A.K., O.P., D.D., E.S., and M.T. performed investigation. Y.A., P.L., and M.H. wrote the original draft. M.H. supervised the study.

## Conflict of Interest

All authors declare no competing financial interests and conflict of interest.

## Supporting information


**Data S1.** Inclusion and exclusion criteria for PD patients and healthy controls in the study.
**Data S2.** Effect of demographic variables on apathy in PD.
**Figure S1.** Multidimensional apathy within the PD sample.
