# Supplementary material for: Differential impact of behavioral, social, and emotional apathy on Parkinson's disease
Source: Ann Clin Transl Neurol. 2018 Aug 14;5(10):1286–91. doi: 10.1002/acn3.626 (PMC6186939; doi:10.1002/acn3.626)
Supplement: Supplementary file 1 — Data S1. Inclusion and exclusion criteria for PD patients and healthy controls in the study. Data S2. Effect of demographic variables on apathy in PD. Figure S1. Multidimensional apathy within the PD sample. [file ACN3-5-1286-s001.docx]

**Supplementary Text 1. Inclusion and exclusion criteria for PD patients and healthy controls in the study.**

Consecutive patients in Neurology clinics were asked if they would like to participate in research. Healthy controls were recruited from a volunteer database. The following inclusion and exclusion criteria were used:

Inclusion criteria

Patients:

- Diagnosed with Parkinson’s disease.
- Male or Female, aged 18 – 80.
- Participant is willing and able to give informed consent for participation in the study.

Healthy volunteers:

- Male or Female, aged 18 – 80
- Participant is willing and able to give informed consent for participation in the study.

Exclusion criteria

Patients:

- Severe cognitive impairment defined as score of <50 on Addenbrooke’s Cognitive Examination – Revised III.
- Presence of concomitant illness (e.g. infection, unstable angina, myocardial infarction or heart, respiratory, renal or liver failure, psychosis) or medication (e.g. antipsychotics) which, based on clinical judgement, would be considered to confound interpretation of cognitive testing.
- History of clinically significant drug or alcohol abuse within 6 months of enrolment.

Healthy volunteers:

- History of clinically significant drug or alcohol abuse within 6 months of enrolment.

**Supplementary Text 2. Effect of demographic variables on apathy in PD.**

To investigate the effects of various demographic variables on apathy in PD, we entered age, sex, cognitive ability (based on Addenbrooke’s Cognitive Examination), years of education, duration of disease and levodopa equivalent dose in a multiple linear regression model to predict levels of apathy. This model was not statistically significant for overall AMI score (*F*(6,66)=1.79, *p*>0.05, *R*^2^=0.14, based on *N*=73 patients for which complete data is available), BA subscale (*F*(6,66)=1.39, *p*>0.05, *R*^2^=0.11), SM subscale (*F*(6,66)=1.69, *p*>0.05, *R*^2^=0.13) and ES subscale (*F*(6,66)=1.46, *p*>0.05, *R*^2^=0.12), suggesting that these variables were not significantly related to apathy.


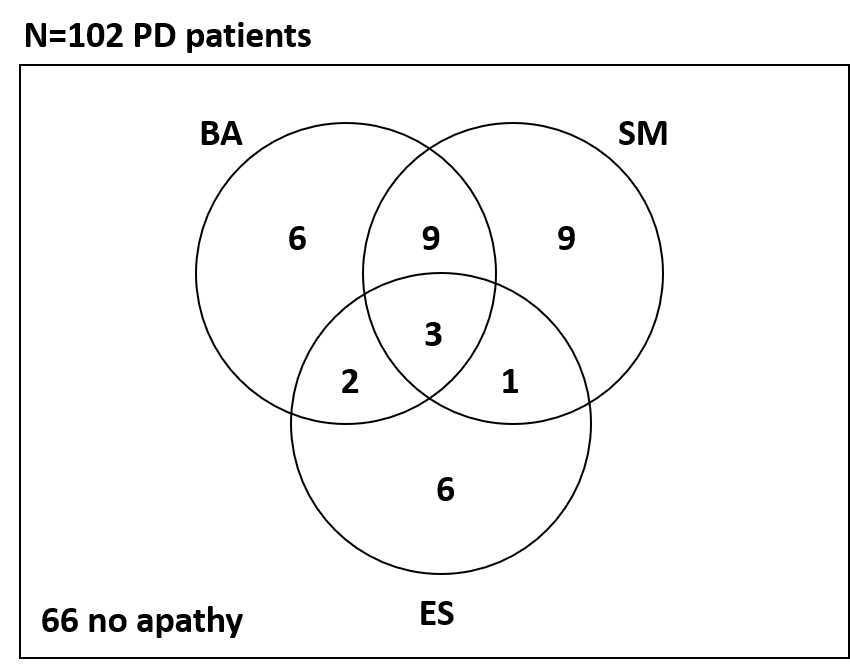


**Supplementary Figure 1. Multidimensional apathy within the PD sample.** Using the AMI cut-off scores derived from Ang et al. (2017)^4^, 36 out of all 102 PD patients were found to be apathetic on at least one subscale. This reflects a prevalence rate of 35.3% that is generally consistent with existing literature^2^. Numbers in the Venn diagram indicate the breakdown of apathetic patients along the AMI subscales: BA (Behavioural Activation), SM (Social Motivation) and ES (Emotional Sensitivity).
